# Ultrasonographic features of cutaneous myxomas in a patient with Carney complex^☆^^☆☆^

**DOI:** 10.1016/j.abd.2020.07.025

**Published:** 2021-09-10

**Authors:** Monica Quintana-Codina, Marina Corral-Forteza, Maribel Iglesias-Sancho, Montse Salleras-Redonnet

**Affiliations:** Department of Dermatology, Hospital Universitari Sagrat Cor - Grupo Quironsalud, Barcelona, Spain

**Keywords:** Carney complex, Myxoma, Skin Diseases, Genetic, Skin neoplasms, Ultrasonography

## Abstract

Carney complex is a rare genodermatosis characterized by cardiac and cutaneous myxomas, among other tumors. In the majority of cases, cutaneous myxomas precede the diagnosis of cardiac myxomas, which are the main cause of death in these patients. Despite the fact that the diagnosis of cutaneous myxomas is histopathological, high-frequency ultrasonography plays an essential role in the differential diagnosis with other cutaneous and subcutaneous tumors. The authors of the present study describe, for the first time in the literature, the ultrasonographic features of both variants of cutaneous myxomas, superficial and subcutaneous, in a patient with a Carney complex.

## Introduction

Carney Complex (CNC) is a rare autosomal dominant neoplasia syndrome characterized by cardiac and cutaneous myxomas, mucocutaneous pigmented lesions (lentigines, blue nevi, and café au lait spots), and multiple endocrine tumors often causing endocrine abnormalities involving various organs.^1^, ^2^, ^3^ The gene PRKAR1A on chromosome 17 (17q22-24) is mutated in more than 70% of cases and about 30% of affected patients carry a *de novo* mutation. This gene codes for the regulatory subunit type I alpha of the protein kinase A enzyme, which is a key component of the cAMP signaling pathway, involved in endocrine tumorigenesis. To date, more than 125 pathogenic inactivating mutations have been reported. For the majority of the PRKAR1A-negative CNC patients, the genetic cause remains unknown.^1^, ^2^, ^4^ In 81% of cases, cutaneous myxomas precede the diagnosis of cardiac myxomas, which are the main cause of death in these patients.^1^, ^5^ Therefore, the detection of this skin tumor should always lead the physician to rule out cardiac involvement. Despite histopathology being the gold standard diagnostic test for cutaneous myxomas, high-frequency ultrasonography (US) can play an essential role in the differential diagnosis of these tumors with other dermic and hypodermic lesions. In this case report, the authors describe for the first time the ultrasonographic features of both superficial and subcutaneous myxomas in a patient with CNC.

## Case report

A 20-year-old man with a personal history of surgical excision of various cutaneous lesions (cysts, lipomas and one myxoma) was referred to the present study’s department for evaluation and treatment of acne. Physical examination revealed facial and labial lentigines, a subcutaneous nodule on the left malar area, as well as multiple pedunculated, velvety, soft, and pink papules located on the trunk.

**Figure 1:**
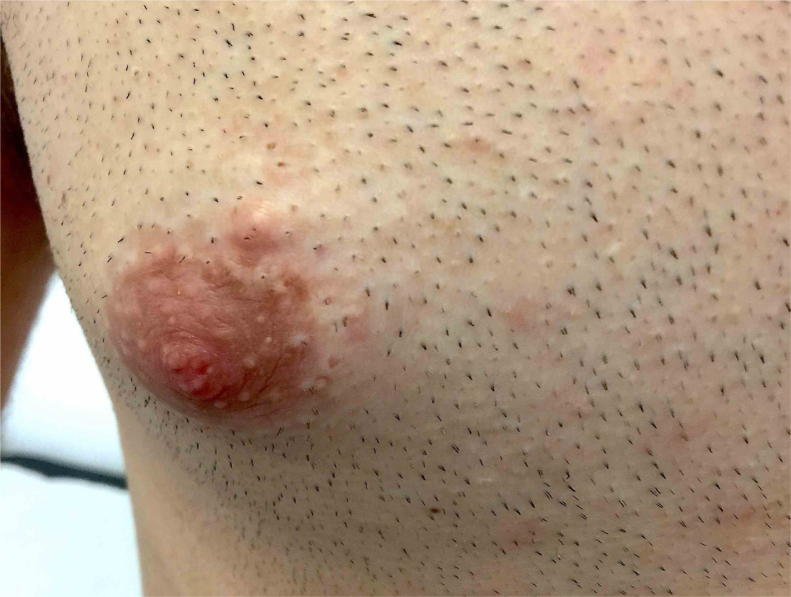
Velvety, soft, and pink papule located on the right periareolar region, corresponding to a superficial cutaneous myxoma.

**Figure 2:**
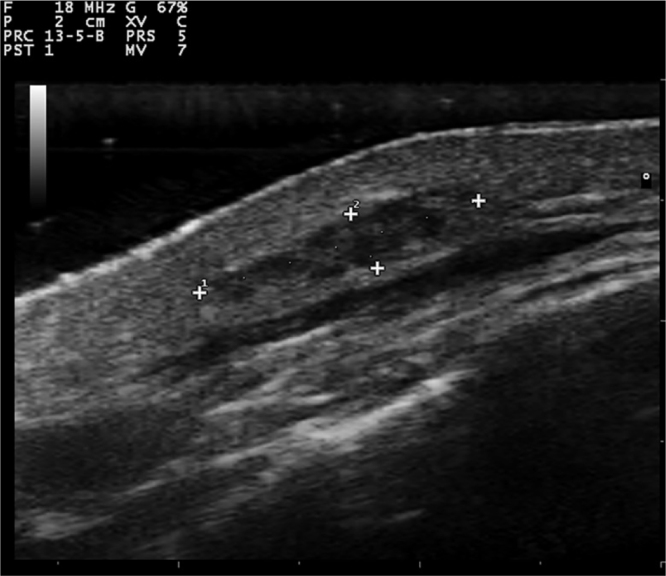
Transverse sonogram (18 MHz) of the subcutaneous myxoma, showing an ill-defined, elongated, heterogeneous, and hypoechoic mass located in the deep dermis and hypodermis.

**Figure 3:**
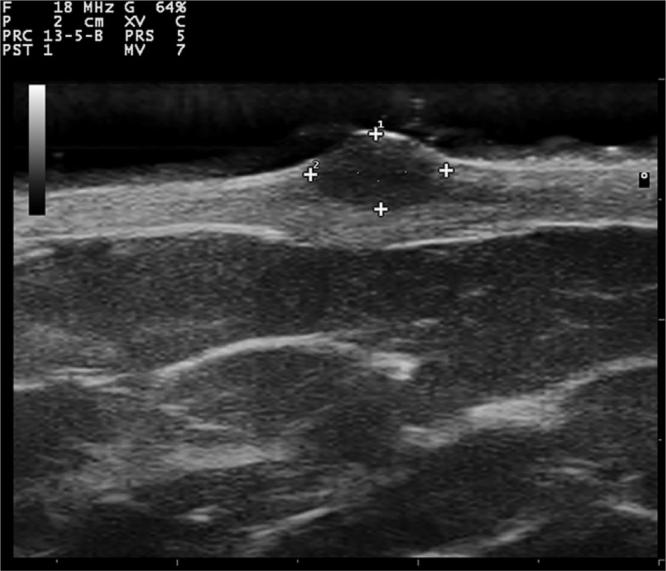
Transverse sonogram (18 MHz) of a superficial myxoma, showing a well-defined, round, homogeneous, and hypoechoic lesion located in the superficial dermis with elevation of the epidermis.

**Figure 4:**
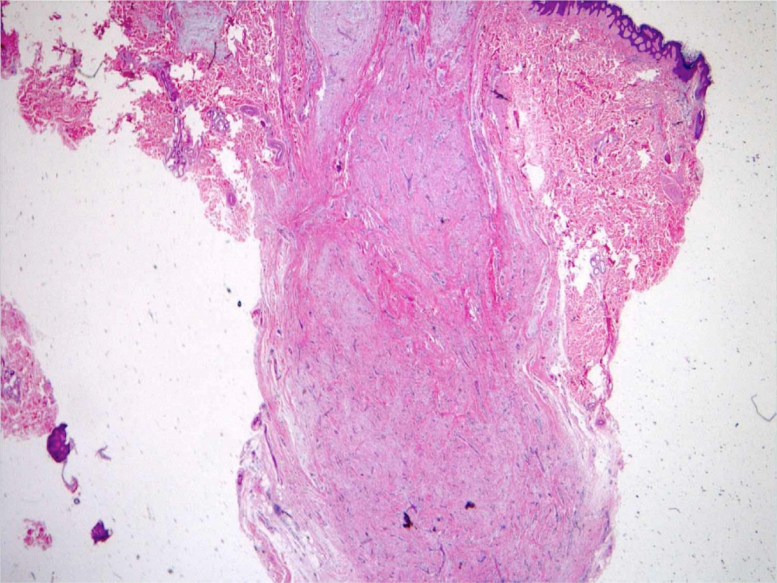
Microphotograph of the superficial myxoma shows follicular adnexal structures amidst the hypocellular myxoid neoplasia and vascular proliferation (Hematoxylin & eosin, ×40).

Gray-scale and color Doppler US examinations of both the subcutaneous nodule and one of the papules located on the right periareolar region (Fig. 1) were performed with a MyLab25Gold scanner equipped with a 10–18 MHz linear array transducer (Esaote, Genoa, Italy). High-frequency US of the subcutaneous malar nodule revealed an ill-defined, elongated, heterogeneous, and hypoechoic mass located in the deep dermis and hypodermis (Fig. 2). On the other hand, the US of the right periareolar papule showed a well-defined, round, homogeneous, and hypoechoic lesion located in the superficial dermis with an elevation of the epidermis (Fig. 3). Color and directional power Doppler US showed no increased vascularity in neither of the two lesions. Surgical removal and histopathology of two papules on the trunk confirmed the diagnosis of cutaneous myxomas (Fig. 4). The malar lesion was not excised for cosmetic reasons.

With the diagnosis of CNC, the following tests were performed: echocardiogram, thyroid and testicular US, 24-h urinary free cortisol, plasma insulin-like growth factor-1, and PRKAR1A sequencing. No alterations were found except for the presence of thyroid colloid nodules and the p.Val164Glu*fs*X5 mutation in the PRKAR1A gene, which was present in the patient but not in his first-grade relatives. Therefore, it was a sporadic case of CNC. The patient is followed regularly in the present study’s department in order to rule out new-onset associations.

## Discussion

Carney complex is a rare genodermatosis with more than 600 cases reported worldwide. Many skin manifestations are numbered among its diagnostic criteria, including cutaneous lentiginosis, cutaneous or mucosal myxomas, and multiple blue nevi. Other skin lesions associated with CNC are *café au lait* spots, melanocytic nevi, lipomas, and angiofibroma. After cutaneous lentigines and blue nevi, cutaneous myxomas are the third most frequent skin manifestation (30%–55% of patients). They usually present as multiple, asymptomatic, sessile, or pedunculated pink papules located commonly on the eyelid, external ear canal, breast nipples, and genitalia. They can be localized in the dermis, or the subcutaneous layer and they measure less than 1 cm in diameter.^1^, ^2^, ^4^

On the other hand, cardiac myxomas are the most common noncutaneous lesions found in CNC (20%–40% of patients). They can appear early in infancy and occur anywhere in the heart. They present with obstructive, constitutional, or embolic phenomena like strokes, heart failure, or sudden death. They are responsible for more than 50% of the mortality of the disease and considering that 81% of patients with cardiac myxomas have previously presented cutaneous myxomas, the existence of the latter should always lead the physician to rule out cardiac involvement.^1^, ^2^, ^4^, ^6^

While cardiac myxomas sonographically present as pedunculated, atrial, hyperechoic masses; ultrasonography of cutaneous myxomas is yet to be described in the literature. The authors have observed that they show different ecographic features depending on their location in the skin. Superficial myxomas present as well-defined, round, homogeneous, and hypoechoic lesions located in the superficial dermis with an elevation of the epidermis. Instead, subcutaneous myxomas appear as ill-defined, elongated, heterogeneous and hypoechoic masses located in the deep dermis and hypodermis. The sonographic differential diagnosis of superficial myxomas should be made with benign skin tumors such as cysts, dermatofibromas, pilomatrixomas, and neurofibromas. Cysts are well-defined, round, hypoechoic, or anechoic lesions and usually present posterior enhancement and bilateral edge shadowing. Dermatofibromas are ill-defined, hypoechoic and heterogeneous lesions. In the US, pilomatrixomas appear as hypoechoic lesions with well-defined margins and typically present multiple small calcifications and posterior shadowing. Color Doppler US generally reveals a single artery that penetrates the lesion and gives rise to multiple intralesional branches.^7^ Neurofibromas are round, oval-shaped, or fusiform hypoechoic lesions that can show a connection with hypoechoic neural tracts. On the other hand, the sonographic differential diagnosis of subcutaneous myxomas should be made with deeper lesions like abscesses or lipomas. In the US, abscesses appear as heterogeneous fluid collections with irregular margins and increased vascular flow on Color Doppler US in case of active inflammation. Lipomas are usually ovoid, hypoechoic, subcutaneous masses with linear hyperechoic horizontal lines within the lesion.

In conclusion, despite the fact that distinctive features have not been identified, the authors believe that high-frequency US is a valuable complementary tool in the diagnosis of cutaneous myxomas in patients suspected of CNC. As far as the authors are concerned, this is the first ultrasonographic description of superficial and subcutaneous myxomas. High-frequency ultrasonography also enables the follow-up of those lesions not tributary to surgical treatment, especially the subcutaneous variant in locations such as the face.

## Financial support

None declared.

## Author’s contributions

Monica Quintana-Codina: Approval of the final version of the manuscript; critical literature review; data collection, analysis, and interpretation; effective participation in research orientation; intellectual participation in propaedeutic and/or therapeutic management of studied cases; manuscript critical review; preparation and writing of the manuscript; study conception and planning.

Marina Corral-Forteza: Approval of the final version of the manuscript; data collection, analysis, and interpretation; effective participation in research orientation; intellectual participation in propaedeutic and/or therapeutic management of studied cases; manuscript critical review; study conception and planning.

Maribel Iglesias-Sancho: Approval of the final version of the manuscript; data collection, analysis, and interpretation; effective participation in research orientation; intellectual participation in propaedeutic and/or therapeutic management of studied cases; manuscript critical review; study conception and planning.

Montse Salleras-Redonnet: Approval of the final version of the manuscript; data collection, analysis, and interpretation; effective participation in research orientation; intellectual participation in propaedeutic and/or therapeutic management of studied cases; manuscript critical review; study conception and planning.

## Conflicts of interest

None declared.
